# Energy scaling and reduction in controlling complex networks

**DOI:** 10.1098/rsos.160064

**Published:** 2016-04-20

**Authors:** Yu-Zhong Chen, Le-Zhi Wang, Wen-Xu Wang, Ying-Cheng Lai

**Affiliations:** 1 School of Electrical, Computer, and Energy Engineering, Arizona State University, Tempe, AZ 85287, USA; 2 Department of Systems Science, School of Management and Center for Complexity Research, Beijing Normal University, Beijing 100875, People’s Republic of China

**Keywords:** complex networks, control, scaling law

## Abstract

Recent works revealed that the energy required to control a complex network depends on the number of driving signals and the energy distribution follows an algebraic scaling law. If one implements control using a small number of drivers, e.g. as determined by the structural controllability theory, there is a high probability that the energy will diverge. We develop a physical theory to explain the scaling behaviour through identification of the fundamental structural elements, the longest control chains (LCCs), that dominate the control energy. Based on the LCCs, we articulate a strategy to drastically reduce the control energy (e.g. in a large number of real-world networks). Owing to their *structural* nature, the LCCs may shed light on energy issues associated with control of nonlinear dynamical networks.

## 1. Introduction

The past 15 years have witnessed tremendous advances in our understanding of complex networked structures in various natural, social and technological systems, as well as the dynamical processes taking place on them [1–6]. Progress has also been made in the area of controlling complex networks, where the ultimate goal is to control nonlinear dynamical processes on complex networks. The interplay between nonlinear dynamics and complex networks makes formulating a general control framework too difficult to be addressed at the present. A reasonable compromise is to study linear controllability [7–23] while retaining the complex network topology in hope to gain insights into the fundamental control issues that can be useful for controlling nonlinear dynamical networks. Some representative results are the following. A key issue was to determine the minimum number of driver nodes required to steer the system from an arbitrarily initial state to an arbitrarily final state in finite time. In this regard, a pioneering approach [10] was to adopt the classic structural controllability theory of Lin [24] to directed complex networks whose structural controllability can be accessed via the maximum matching algorithm [25–27]. The effects of the density of in/out degree nodes were incorporated into the structural controllability framework [17], which was also applied to protein interaction networks [19]. Based on the classic Popov–Belevitch–Hautus (PBH) rank condition [28], an exact controllability framework was developed [16,20].

An issue of physical importance is the energy required to control a complex network. The energy bounds were first obtained for specific classes of networks [13]. For example, if the network adjacency matrix is positive definite, the lower bound of the energy approaches a constant but if the matrix is semi-positive definite, the lower bound scales algebraically with the control time. In these cases, the upper bound of the energy can still diverge. Quite recently, it was found [22,23] that under certain conditions (e.g. setting the number of controllers to one or the entire network size), for scale-free networks the actual control energy follows a power-law (algebraic) distribution with respect to uncertainties in the selection of the target state. In the extreme case where the structural controllability theory stipulates, mathematically, that a single controller be sufficient to control the entire network, there is a high probability for the energy to diverge. While the results provide insights into the feasibility of controlling complex networks, the physical underpinning for the algebraic energy distribution and energy divergence needs to be understood, which is the main purpose of our study.

In this paper, we uncover the general mechanism responsible for the control energy, without any restrictions on the number of controllers, topology and the target state. Our main result is the following. We find that, for any given complex network, the fundamental entities responsible for the control energy possess a chain structure, which we call the longest control chains (LCCs). (LCCs are conceptually different from control signal paths (CSPs), or stems [10,18]—see §2.3 for explanations.) Identification of LCCs enables us to obtain a physical understanding of the energy distribution, providing an explanation for our numerically discovered phenomenon that energy divergence is prevalent in real-world networks. The understanding also allows us to articulate effective strategies to drastically reduce the control energy (e.g. by many orders of magnitude). Because LCCs are a structural concept, we expect it to be useful for addressing the energy issue associated with control of nonlinear networks.

## 2. Results

### 2.1. Control energy distribution

We use the standard setting of network controllability [7,10,16]:

2.1 $$\dot{\text{x}}=A\text{x}+B\text{u},$$

where **x**=[*x*_1_(*t*),…,*x*_*N*_(*t*)]^T^ is the state variable of the whole system, vector **u**=[*u*_1_(*t*),…,*u*_*M*_(*t*)]^T^ is the control input or the set of control signals, *A* is the *N*×*N* adjacency matrix of the network, and *B* is the *N*×*N*_D_ control matrix specifying the set of ‘driver’ nodes [10]. The goal of the structural and exact controllability theories is to determine the minimum number of driver nodes, *N*_D_, for networks of various topologies. With the input driving signal **u**_*t*_, the control energy is defined as

2.2 $$E(t_{f})=\int_{0}^{t_{f}}{\text{u}}_{t}^{T}\cdot {\text{u}}_{t}\;dt.$$

In the standard linear systems theory [29], optimal control can be achieved to minimize the energy in the functional space of the control input signal **u**_*t*_. The optimal control signal is

2.3 $${\text{u}}_{t}=B^{T}\cdot e^{A^{T}(t_{f}-t)}\cdot W^{-1}\cdot ({\text{x}}_{t_{f}}-e^{At_{f}}\cdot {\text{x}}_{0}),$$

where $W\equiv \int_{t_{0}}^{t_{f}}e^{A\tau }B\cdot B^{T}\cdot e^{A^{T}\tau }d\tau$ is the positive-definite and symmetric Gramian matrix.

**Figure 1.**
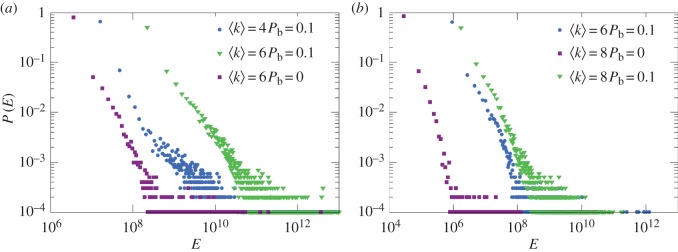
Energy distributions for (*a*) ER random and (*b*) BA scale-free networks. Each distribution is obtained from an ensemble of 10 000 networks. For most networks in the ensemble, the control energies diverge for values of the probability *P*_b_ or the average degree 〈*k*〉 larger than the ones shown in the figure. The initial states **x**_0_ and the final states **x**_*t*_f__ are randomly chosen, with the control time set to *t*_f_=1.

We use the Erdos and Rényi (ER) type of directed random networks [30,31] and the Barabási–Albert (BA) type of directed scale-free networks [2] with a parameter *P*_*b*_. Specifically, for a pair of nodes *i* and *j* with a link, the probability that it points from the smaller degree to the larger degree nodes is *P*_b_, and 1−*P*_b_ is the probability that the link points in the opposite direction (if both nodes have the same degree, the link direction is chosen randomly). We implement the maximum matching algorithm [10] to obtain the control matrix *B* and calculate the minimum energy for any given control time *t*_f_. For an ensemble of randomly realized network configurations with identical structural properties, the control energy *E* can be regarded as a random variable. We find that, for a vast majority of the networks in the ensemble, the required control energy is enormous and tends to diverge. For the cases where the energy can be reasonably computed, it follows an algebraic distribution with fat tails, as shown in figure 1 with the scaling exponent of about 1.5, regardless of the network type and size.

### 2.2. Structural determinants of control energy and energy distribution

We develop a physical understanding of the large control energy required and also the algebraic scaling behaviour in the energy distribution. To gain insights, we first consider a simple model: a unidirectional, one-dimensional string network, for which an analytic estimate of the control energy can be obtained [23] as

**Figure 2.**
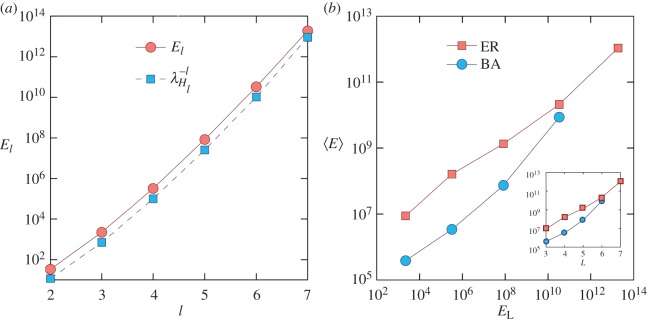
(*a*) For a one-dimensional chain network of length *l*, energy *E*_*l*_ and λ^−1^_*H*_*l*__ versus *l*. (*b*) Correlation between 〈*E*〉, the average of control energy for networks with the same LCC length, and *E*_L_, the energy of an LCC of length *D*_C_=*L* (*L*=3, 4, 5, 6 and 7 for ER and *L*=3, 4, 5 and 6 for BA networks), calculated from ensembles of 10 000 networks. The inset in (*b*) shows the corresponding 〈*E*〉 versus the LCC length *L* for BA and ER networks.

2.4 $$E_{l}\approx \lambda_{H_{l}}^{-1},$$

where *l* is the chain length (the number of nodes on the string) and λ_*H*_*l*__ is the smallest eigenvalue of the underlying *H*-matrix, denoted by *H*_*l*_, which is related to the Gramian matrix by *H*≡*e*^−*At*_f_^*We*^−*A*^T^*t*_f_^. Numerical verification of equation (2.4) is presented in figure 2*a*. Although equation (2.4) is obtained for a simple one-dimensional chain network, we find numerically and analytically that it also holds for random and scale-free network topologies.

A brief derivation of equation (2.4) is as follows. The control energy required by the system can be expressed as [13]

2.5 $$E(t_{f})={\text{x}}_{0}^{T}\cdot H^{-1}\cdot {\text{x}}_{0},$$

where **x**_0_ is the initial state of the system. The matrix *H* is positive definite and symmetric with its inverse satisfying *H*^−1^=*QΛQ*^T^, where *Λ* and *Q* are the corresponding eigenvalue and eigenvector matrices, respectively. Thus, $\lambda_{H}^{-1}$ is the largest eigenvalue of *H*^−1^ with λ_*H*_ denoting the smallest eigenvalue of *H*. Numerically, we find that $\lambda_{H}^{-1}$ is dramatically larger than any other eigenvalue. We have

2.6 $$\begin{array}{rl} E(t_{f}) & ={\text{x}}_{0}^{T}Q\Lambda Q^{T}{\text{x}}_{0}=\sum_{i=1}^{N}\lambda_{i}{\left({\text{q}}_{i}^{T}\cdot {\text{x}}_{0}\right)}^{2}\\ & \approx \lambda_{H}^{-1}{\left({\text{q}}_{1}^{T}\cdot {\text{x}}_{0}\right)}^{2}, \end{array}$$

where **q**_*i*_ is the *i*th column of *Q*. If the initial state **x**_0_ is chosen to satisfy ${\text{q}}_{1}^{T}\cdot {\text{x}}_{0}=1$, we obtain $E(t_{f})\approx \lambda_{H}^{-1}$. We find numerically that a random choice of **x**_0_ does not change *E*(*t*_f_)’s order of magnitude. A detailed derivation can be found in [23].

We see from figure 2*a*, that the energy required for control tends to increase exponentially with the chain length, indicating that even for a simple one-dimensional chain network of limited length, such as *l*=7, the required control energy can be unbearably large. The exponential behaviour holds for complex networks as well, as shown in the inset of figure 2*b* for ER random and BA scale-free networks. In fact, figure 2*b* indicates a strong positive correlation between the average control energy for the network and *E*_L_, the energy required to control the LCC (to be described below) embedded in the network.

### 2.3. Concepts of control signal paths and longest control chains

**Figure 3.**
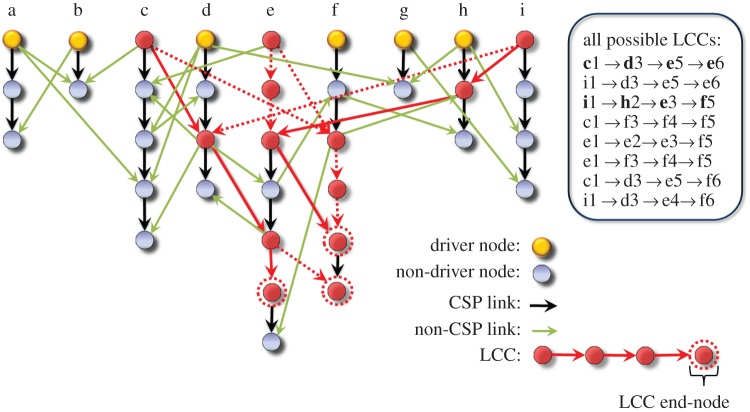
Schematic of CSPs (stems) and LCCs of a network. There are *N*_D_=9 CSPs, which are aligned vertically and labelled as *a* to *i*. CSP (or non-CSP) links are displayed in black (or green). In this example, the length of the LCCs is 4. Typically, a control chain may contain nodes belonging to multiple CSPs. Two LCCs sharing no common nodes are marked by the red nodes and the solid red arrows. Links belonging to other LCCs are marked by red dashed arrows. Each node is specified using its CSP label and its position along the CSP sequentially from top to bottom. Eight LCCs in the network converge to only three end-nodes, *e*6, *f*5 and *f*6 (marked by red dashed circles), leading to LCC degeneracy *m*=3. Note that the stems [10] (or CSPs) are originated from the driver nodes and are obtained through maximal matching.

In a networked system, control signal and energy originated from the driver nodes travel through one-dimensional-string-like paths towards each of the non-driver nodes. As discussed, identifying maximum matching so that the network is deemed structurally controllable does not guarantee convergent control energy. When maximum matching is found, one can divide the whole network into *N*_D_ CSPs, namely, *N*_D_ *stems* [10,18], each being a unidirectional one-dimensional string led by a driver node that passes the control signal onto every node along the path, illustrated as the vertical paths in figure 3. CSPs thus provide a picture indicating how the signals from the *N*_D_ external control inputs reach every node in the network to ensure full control (in the sense of structural controllability).

How does the control energy flow through the network? To address this question, we distinguish two types of links: one along and another between the CSPs, as shown in figure 3. It may seem that the latter links are less important as the control signal propagates along the former set of links. However, owing to coupling, a node’s dynamical state will affect all its nearest neighbours’ states which, in turn, will affect the states of their neighbours, and so on. In principle, any driver node is connected with nodes both along and outside its CSP. Correspondingly, an arbitrary node in the network is influenced by every driver node, directly through the CSP to which it belongs, or indirectly through the CSPs that it does not sit on. Intuitively, the ability of a driver node to influence a node becomes weaker as the distance between them is increased. In order to control a distant node, exponentially increased energy from the driver is needed. The chain starting from a driver node and ending at a non-driver node along their shortest path is effectively a control chain. Intuitively, control energy flows through the control chains, while the control signal is propagated along the CSPs. We define the length of the longest control chain (LCC), *D*_C_, as the *control diameter* of the network, as shown in figure 3.

To find the LCCs in a network, we first use the maximum matching algorithm to find all the driver nodes. We then identify the shortest paths from each of the driver nodes to each of the non-driver nodes. Finally, we pick out the longest such shortest paths as LCCs. The computational complexity of finding the LCCs are that associated with the maximum matching algorithm plus searching for the longest shortest paths, which is feasible for large networks. There can be multiple LCCs. The node at the end of an LCC is most difficult to be controlled in the sense that the largest amount of control energy is required. The number *m* of such end nodes dictates the degeneracy (multiplicity) of LCCs. An example is shown in figure 3, where we see that, although there are multiple LCCs, their ends converge to only three nodes, leading to *m*=3. Since the energy required to control a one-dimensional chain grows exponentially with its length in such a way that even one unit of increase in the length can amplify the energy by orders of magnitude (figure 2*a*), the energy associated with any chain shorter than the LCC can typically be several orders of magnitude smaller than that with the LCC. Thus, the total energy is dominated by the LCCs. Owing to the typically low value of *m*, a single LCC essentially dictates the energy required to control the whole chain system. This is true especially for networks with long LCCs. Intuitively, the probability to form long LCCs is small. Accordingly, a longer LCC tends to have smaller value of degeneracy *m*. As a result, the longest LCCs have almost no degeneracy (*m*=1) so that they effectively rule the control energy of the whole network.

In the structural controllability theory, a network is deemed more structurally controllable if *N*_D_ is smaller [10]. However, as the number of driver nodes is reduced, the length of the chain of nodes that each controller drives on average must increase, leading to an increase in the LLC length and accordingly an exponential growth in the control energy. In the ‘optimal’ case of structural controllability of *N*_D_=1, the LLC length will be maximized, leading to unrealistically large control energy that prevents us from achieving actual control of the system.

Taken together, with respect to the previously defined concept of stem [10,18], we emphasize that a stem is a path that propagates control signal from the input in the absence of a feedback loop (or a circle), and each such path is determined by maximum matching, which allows a node to control at most one of its immediate neighbours. Thus, a stem is in fact a CSP, which is quite different from the concept of an LCC. Particularly, for a driver node and a non-driver node, a control chain is the shortest path between them. For a set of driver nodes and all non-driver nodes in the entire network, an LCC is simply the longest control chain. While control signals propagate through the CSPs, the energy may not flow along the same paths due to the interactions among the nearest neighbours via the physical connections. For example, the state change of a node can lead to state changes of all its nearest neighbours through the energy exchange between this node and all its neighbours. That is, control energy flows through the LCCs, not CSPs.

### 2.4. Control energy reduction scheme

Our finding of the LCC skeleton suggests a method to significantly reduce the control energy. A straightforward solution is to break the LCCs with *redundant* controllers beyond those obtained via maximum matching along the LCCs. Adding a redundant control input at the *i*th node of a unidirectional one-dimensional chain of length *l* breaks it into two shorter sub-chains of length *i*−1 and *l*−*i*+1. Roughly, the control energy is the sum of energies required to control the two shorter components, which is dominated by energy associated with the longer one owing to the exponential dependence of the energy on the chain length. For *i*≈*l*/2, the length of the longer part is minimized. In simulations, applying a single redundant control input presents an extremely efficient energy reduction strategy for one-dimensional chains: several orders of magnitude of reduction in the energy can be achieved. As a realistic physical example, we considered a bidirectional cascading parallel R-C circuit of seven units, which is effectively a one-dimensional chain of seven nodes with a self-loop at each node. For this system, the redundant control input can be realized by inducing external current input into a capacitor. A reduction in the joule energy of nearly 10 orders of magnitude is achieved [23].

For a complex network, the strategy of adding a redundant control at the middle of each LCC dramatically outperforms the strategy of randomly adding an identical number of control inputs. Treating the amount of the normalized energy reduction Δ*E*/*E* as a random variable across the ensemble of networks, we obtain a monotonously increasing distribution function *P*(Δ*E*/*E*), reflecting the effect of energy reduction. We find that high (low) Δ*E*/*E* values are more (less) probable under LCC-breaking strategy when compared with the random strategy. For networks with longer LCCs, the strategy works more effectively. For example, for networks with *D*_C_=5, nearly 40% of the networks reach Δ*E*/*E*≈1, but the same reduction can be achieved only for 2% of the networks via the random strategy.

**Figure 4.**
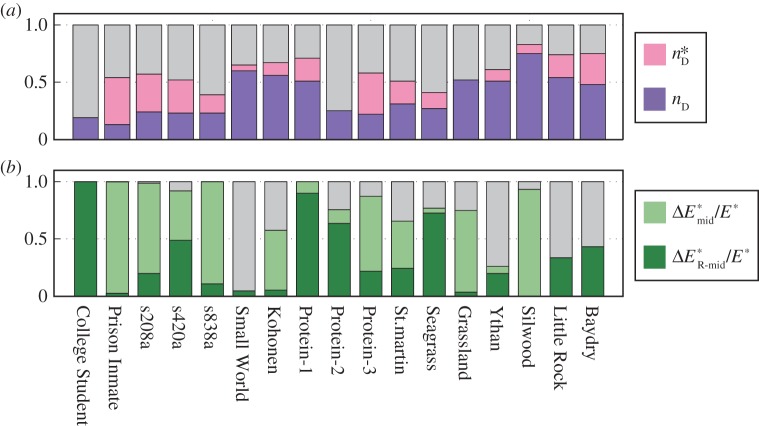
For the 17 real-world networks studied in [10], (*a*) densities of the original driver nodes *n*_D_ (dark purple) and of the augmented controls $n_{D}^{⋆}$ (light purple). (*b*) Normalized energy reduction $\Delta E_{\mathrm{mid}}^{⋆}/E^{⋆}=(E^{⋆}-E_{\mathrm{mid}}^{⋆})/E^{⋆}$ (light green) when an additional control signal is added to the middle of each LCC and normalized energy reduction $\Delta E_{R\text{-}mid}^{⋆}/E^{⋆}=(E^{⋆}-E_{R\text{-}mid}^{⋆})/E^{⋆}$ (dark green) for the case where the same number of control signals are randomly added into each network. All coloured bars start from 0. For the ‘Silwood’ network, the energy with randomly added control signals is higher than the corresponding *E*^★^ (the negative $\Delta E_{R\text{-}mid}^{⋆}$ is not shown in the figure). For the light green bars, the reduced control energy $E_{\mathrm{mid}}^{⋆}$ is several orders of magnitude smaller than *E*^★^. The bars with more gray portion than the light green potions are for the networks with relatively low values of *E*^★^ and *D*_C_, for which energy reduction is not necessary.

For a large number of real-world networks, the mathematical structural controllability theory stipulates that some of them are controllable with a few driving signals [10]. We find that most of these networks (15 out of 17) require unrealistically high energies due mainly to their long LCCs. Strikingly, even with unlimited energy supply, the number of driver nodes as determined by the maximum matching algorithm is generally insufficient to fully control the whole system, where there exists a number *M*^★^ of nodes that never converge to their target states. These observations lead to the speculation that, in order to fully control a realistic network, significantly more driver nodes are needed than those identified by maximum matching. For example, *N*^★^_D_=*N*_D_+*M*^★^ driver nodes should be deployed to gain full control of the system, as shown in figure 4*a* for *n*_D_ and $n_{D}^{⋆}=N_{D}^{⋆}/N$ for the 17 real-world networks. Our energy reduction strategy is remarkably effective on the real-world networks with large LCCs, as shown in figure 4*b*. The quantity $E_{\mathrm{mid}}^{⋆}$ is the control energy of a real-world network when a redundant control input is applied at the middle of each LCC, while $E_{R\text{-}mid}^{⋆}$ denotes the energy when the same number of redundant control inputs are randomly applied to the network. For each of the real-world networks with unrealistically large energy requirement, the reduced control energy $E_{\mathrm{mid}}^{⋆}$ is several orders of magnitude lower than the original energy *E*^★^—the control energy with *M*^★^ augmented driver nodes without any LCC-breaking control input. The energy reduction due to random control signal augmentation is again much less effective in most cases, giving further evidence that the control energy of real-world networks is generally determined by their LCCs.

To the best of our knowledge, the failure to converge to the target state with limitless energy supply has not been observed for any modelled networks: it only occurs in empirical (real-world) networks. It may be caused by some atypical structural properties. For example, for some networks, the uncontrollable nodes form self-loops but without any incoming links. For some other networks with an unusually high average degree, an ill-conditioned Gramian matrix can arise, prohibiting the system from converging to the target state. However, such uncontrollable cases (even with infinite supply of energy) are not expected to be generic.

## 3. Discussion

We fill a significant knowledge gap in the extremely active field of controlling complex networks by developing a physical understanding of the recently discovered phenomena of algebraic distribution and divergence of the required control energy. This is achieved through identification of LCCs, the fundamental structure embedded in the network that dominantly determines the energy. The understanding leads naturally to an optimization strategy to significantly reduce the control energy in real-world networks: increasing the number of controllers slightly by placing redundant control signals (beyond the number determined by the structural-controllability theory) along the LCCs. At present, the issue of control energy can be addressed only in the linear controllability framework, but we believe that the LCCs, due to their structural origin, can find counterparts in formulating theories of controllability and observability for nonlinear dynamical networks [9,21,32,33], a largely open problem deserving much attention and great research effort.

Our results that the LCCs essentially determine the network control energy are consistent with the finding in [34]. In particular, for a one-dimensional unidirectional chain with exactly one control input, its control energy has the standard definition that leads to the mathematical conclusion of energy divergence for long chains, while the finding that anisotropic networks can be readily controlled was not obtained under exactly the same setting. The dependence of a network’s control energy on its LCCs can be further demonstrated via our energy reduction schemes, effective for both modelled and real-world complex networks.

## 4. Methods

### 4.1. Longest control chain and control energy distribution

The construction illustrated in figure 3 provides a structural profile to estimate the control energy. In particular, a network can be viewed as consisting of a set of control chains, and the total energy *E* required has two components: *E*^(1)^, the sum of energies associated with all control chains, and *E*^(2)^, the interaction energies among the chains, defined as *E*−*E*^(1)^. The control chains are approximately independent of each other so that *E*^(2)^ is negligible as compared with *E*^(1)^. Each control chain is effectively a one-dimensional string. Owing to the exponential increase in the energy with the chain length, *E*^(1)^ can be regarded as the sum of control energies associated with the set of LCCs. The required energy to control the full network can thus be approximated as

4.1 $$E=E^{\left(1\right)}+E^{\left(2\right)}\approx E^{\left(1\right)}\approx m\cdot E_{D_{C}},$$

where *E*_*D*_C__ denotes the energy required to control an LCC of length *D*_C_, and *m* denotes its degeneracy (multiplicity), as shown in figure 3. Reasoning from an alternative standpoint, an arbitrary combination of *D*_C_ and *m*, which we call an LCC *skeleton*, effectively represents a network, and the entire network ensemble is equivalent to all the possible LCC skeletons according to a joint probability function *P*(*D*_C_,*m*). The energy distribution for the original network can be characterized by the distribution of the energy required to control the LCC skeleton in the ensemble. Through simulations, we find that the marginal distribution of *D*_C_, *P*_*D*_C__(*D*_C_), decays approximately exponentially:

4.2 $$P_{D_{C}}(D_{C})=a\cdot e^{-b\cdot D_{C}},$$

where *a* and *b* are positive constants. Using

4.3 $$E_{D_{C}}\approx A\cdot e^{B\cdot D_{C}},$$

we obtain

4.4 $$D_{C}\approx \left(\frac{1}{B}\right)\ln\left(\frac{E_{D_{C}}}{A}\right),$$

where *A* and *B* are positive constants, and consequently the distribution of *E*_*D*_C__ as

4.5 $$\begin{array}{rl} P(E_{D_{C}}) & =P_{D_{C}}\left(\frac{\ln\left(E_{D_{C}}/A\right)}{B}\right)\cdot \left|\frac{d\ln\left(E_{D_{C}}/A\right)}{dE_{D_{C}}}\right|\\ & \approx \frac{aA^{b/B}}{B}\cdot E_{D_{C}}^{-(1+b/B)}. \end{array}$$

The marginal distribution of *m* for networks with *D*_C_>2 also exhibits an exponential decay:

4.6 $$P_{m}(m)=c\cdot e^{-g\cdot m},$$

where *c* and *g* are positive constants. Note that *D*_C_ and *m* are uncorrelated, since a positive correlation would imply that the number of LCCs increases with their length, which is unphysical, and a negative correlation would lead to an exponential divergence of either *P*_*D*_C__(*D*_C_) or *P*_*m*_(*m*). We thus have

4.7 $$P(D_{C},m)\approx P_{D_{C}}(D_{C})\cdot P_{m}(m)$$

and

4.8 $$P(E_{D_{C}},m)\approx P_{D_{C}}(E_{D_{C}})\cdot P_{m}(m).$$

As a result, we obtain the following cumulative energy distribution:

4.9 $$\begin{array}{rl} F_{E}(E) & =P(m\cdot E_{D_{C}}<E)=\int_{0}^{\infty }\left[\int_{0}^{E/E_{D_{C}}}P(E_{D_{C}},m)\cdot dm\right]dE_{D_{C}}\\ & =\int_{0}^{\infty }\left[\int_{0}^{E/E_{D_{C}}}P_{D_{C}}(E_{D_{C}})\cdot P_{m}(m)\cdot dm\right]dE_{D_{C}}\\ & \approx \frac{caA^{b/B}}{gB}\cdot \left\{-\frac{b}{B}-\left[\Gamma \left(\frac{b}{B}\right)-\Gamma \left(\frac{b}{B},gE\right)\right]\cdot {\left(gE\right)}^{-b/B}\right\}, \end{array}$$

where *Γ*(*b*/*B*) and *Γ*(*b*/*B*,*gE*) are the Gamma and incomplete Gamma functions, respectively. The distribution of *E* can then be expressed as

4.10 $$P_{E}(E)=\frac{dF_{E}(E)}{dE}\approx \frac{caA^{b/B}}{gB}\cdot \left\{-\frac{e^{-gE}}{E}+\left[\Gamma \left(\frac{b}{B}\right)-\Gamma \left(\frac{b}{B},gE\right)\right]\cdot {\left(gE\right)}^{-(1+b/B)}\right\},$$

where −*e*^−*gE*^/*E*≈0 due to the typically large value of *E*. Since numerically the difference between the two Gamma functions is approximately constant:

4.11 $$h_{\Gamma }\equiv \Gamma \left(\frac{b}{B}\right)-\Gamma \left(\frac{b}{B},\;gE\right)\approx 1.7,$$

*P*_*E*_(*E*) can be simplified as

4.12 $$P_{E}(E)\approx C\cdot E^{-(1+b/B)},$$

*providing a theoretical explanation for the algebraic energy distribution*, where

$$C=\left[\frac{caA^{b/B}}{gB}\cdot g^{-(2+b/B)}\cdot h_{\Gamma }\right]$$

is a positive constant. Numerically, we have *B*≈2 and *b*≈1. Accordingly, the theoretical estimate of the scaling exponent is 1+*b*/*B*≈1.5, which is consistent with the numerical value. The nearly identical exponent in the distribution of *E*_*D*_C__ indicates that the role of the LCC degeneracy *m* is insignificant in the control energy and its distribution. It is the combination of the exponential decay in the distribution of the control diameter and the exponential increase in the energy required to control LCCs with their length which gives rise to the power-law energy distribution of the LCCs and ultimately leads to the algebraic distribution in the actual energy required to control the original network.

We remark that, since the probability *P*_*m*_(*m*) has an exponential dependence on *m* and does not explicitly contain the energy *E*_*D*_C__, the summation in *m* from 1 to *E*/*E*_*D*_C__ is a finite geometric sum, which differs from the integral in only a constant coefficient and does not affect the algebraic scaling exponent 1+*b*/*B*. In our analysis, the reason to treat *m* as a real valued number is that the relation *E*≈*mE*_*D*_C__ holds only in the order-of-magnitude sense, implying that *E*/*E*_*D*_C__ is typically not an integer. The limits m→0 and $E_{D_{C}}\to \infty$ correspond to the rare case where all nodes in the network belong to one single LCC.

If we treat *m* (1≤*m*≤*E*/*E*_*D*_C__) as an integer and accordingly set the integral upper bound of *E*_*D*_C__ as *E*, the cumulative energy distribution function will have a similar form to equation (4.9) with only a small difference in the constant coefficient. This would not have any significant effect on the probability density function *P*_*E*_(*E*) in equation (4.12). Numerically, *E* is typically a large number in the order of at least 10^10^. As a result, the numerical difference caused by the integral upper bound is negligible.

### 4.2. Double-chain interaction

Our analysis of the LCC-skeleton predicts power-law distribution of the required energy for practically controllable networks, which agrees qualitatively with numerics. However, interaction energy *E*^(2)^ among the coexisting chains is ignored. In a physical system, interactions among the basic components can play an important role in determining the system’s properties. To obtain a more accurate estimate of the behaviours of the control energy, we need to include the interactions among the chains. The necessity is further justified as there are discrepancies between the actual control energy and that from the LCC-skeleton, as exemplified in figure 2*b*. Thus, in order to reproduce the numerically obtained energy distributions, we must incorporate the interactions among the LCCs into the model. However, including the interactions makes analysis difficult, as there are typically a large number of interacting pairs of chains. To gain insight into the role played by the interactions, it is useful to focus on the relatively simple case of two interacting chains.

**Figure 5.**
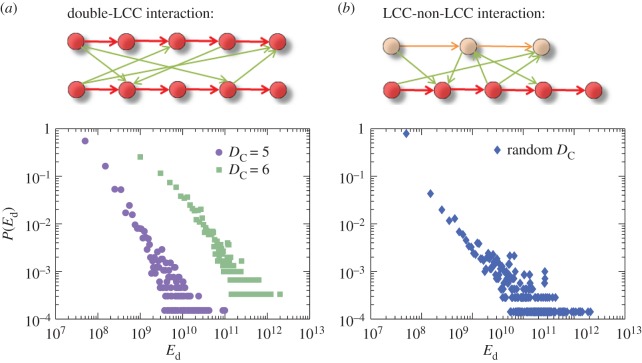
Distribution of control energy in a double-chain interaction models. (*a*) Two chains of *D*_C_=5 and *D*_C_=6, where the upper panel is a schematic of two LCCs of identical length *D*_C_=5 interacting with each other via some random links between them. (*b*) Two chains with their lengths randomly chosen from 3 to 6, where the upper panel shows the case of two interacting chains of length 5 and 3. The longer chain plays the role of LCC, while the shorter chain is a non-LCC.

Our double-chain interaction model is constructed, as follows. Consider two identical unidirectional chains, denoted by *C*1 and *C*2, each of length *D*_C_. Every node in *C*1 connects with every node in *C*2 with probability *p*, all links between the two chains are unidirectional. A link points to *C*2 from *C*1 with probability $p_{1\to 2}$ and the probability for a link in the opposite direction is $p_{2\to 1}=1-p_{1\to 2}$. By changing the connection rate *p* and the directional bias $p_{1\to 2}$, we can simulate and characterize various interaction patterns between the two chains. To be concrete, we generate an ensemble of 10 000 interacting double-chain networks, each with 2⋅*D*_C_ nodes and multiple randomized inter-chain links as determined by the parameters *p* and $p_{1\to 2}$. As shown in figure 5*a*, the distribution of the control energy displays a remarkable similarity to that for random networks, in that a power-law scaling behaviour emerges with the exponent about 1.5. The power-law distribution holds robustly with respect to variations in the parameters *p* and $p_{1\to 2}$, and the change in the magnitude of the energy due to small variations in the parameter values is insignificant when compared with that caused by a change in the length of the LCC. In addition, to reveal the role of the interaction between an LCC and a non-LCC chain in the control energy, we randomly pick their lengths from [3,6] with equal probability, where the longer chain acts as an LCC. Again, we observe a strong similarity between the energy distributions from random networks and from this model, as shown in figure 5*b*, suggesting a universal pattern followed by pair interactions, regardless of the length of the chains. In particular, interactions between two chains, LCC or not, have a similar effect on the control-energy distribution. These results indicate that the double-chain interaction model captures the essential physical ingredients of the energy distribution in controlling complex networks.

## References

[1] Strogatz SH 2001 Exploring complex networks. *Nature* 410, 268–276. (doi:10.1038/35065725) 11258382 10.1038/35065725

[2] Albert R, Barabási AL 2002 Statistical mechanics of complex networks. *Rev. Mod. Phys.* 74, 47–97. (doi:10.1103/RevModPhys.74.47)

[3] Mendes JFF, Dorogovtsev SN, Ioffe AF 2003 *Evolution of networks: from biological nets to the internet and the WWW*, 1st edn Oxford UK: Oxford University Press.

[4] Pastor-Satorras R, Vespignani A 2004 *Evolution and structure of the internet: a statistical physics approach*, 1st edn Cambridge UK: Cambridge University Press.

[5] Barrat A, Barthélemy M, Vespignani A 2008 *Dynamical processes on complex networks*, 1st edn Cambridge UK: Cambridge University Press.

[6] Newman MEJ 2010 *Networks: an introduction*, 1st edn Oxford, UK: Oxford University Press.

[7] Lombardi A, Hörnquist M 2007 Controllability analysis of networks. *Phys. Rev. E* 75, 056110 (doi:10.1103/PhysRevE.75.056110) 10.1103/PhysRevE.75.056110 17677136

[8] Liu B, Chu T, Wang L, Xie G 2008 Controllability of a leader-follower dynamic network with switching topology. *IEEE Trans. Automat. Contr.* 53, 1009–1013. (doi:10.1109/TAC.2008.919548)

[9] Rahmani A, Ji M, Mesbahi M, Egerstedt M 2009 Controllability of multi-agent systems from a graph-theoretic perspective. *SIAM J. Contr. Optim.* 48, 162–186. (doi:10.1137/060674909)

[10] Liu YY, Slotine JJ, Barabási AL 2011 Controllability of complex networks. *Nature* 473, 167–173. (doi:10.1038/nature10011) 21562557 10.1038/nature10011

[11] Wang WX, Ni X, Lai YC, Grebogi C 2011 Optimizing controllability of complex networks by small structural perturbations. *Phys. Rev. E* 85, 026115 (doi:10.1103/PhysRevE.85.026115) 10.1103/PhysRevE.85.026115 22463287

[12] Nacher JC, Akutsu T 2012 Dominating scale-free networks with variable scaling exponent: heterogeneous networks are not difficult to control. *New J. Phys.* 14, 073005 (doi:10.1088/1367-2630/14/7/073005)

[13] Yan G, Ren J, Lai YC, Lai CH, Li B 2012 Controlling complex networks: How much energy is needed? *Phys. Rev. Lett.* 108, 218703 (doi:10.1103/PhysRevLett.108.218703) 23003312 10.1103/PhysRevLett.108.218703

[14] Nepusz T, Vicsek T 2012 Controlling edge dynamics in complex networks. *Nat. Phys.* 8, 568–573. (doi:10.1038/nphys2327)

[15] Liu YY, Slotine JJ, Barabási AL 2013 Observability of complex systems. *Proc. Natl Acad. Sci. USA* 110, 2460–2465. (doi:10.1073/pnas.1215508110) 23359701 10.1073/pnas.1215508110 PMC3574950

[16] Yuan ZZ, Zhao C, Di ZR, Wang WX, Lai YC 2013 Exact controllability of complex networks. *Nat. Commun.* 4, 2447 (doi:10.1038/ncomms3447) 24025746 10.1038/ncomms3447 PMC3945876

[17] Menichetti G, Dall’Asta L, Bianconi G 2014 Network controllability is determined by the density of low in-degree and out-degree nodes. *Phys. Rev. Lett.* 113, 078701 (doi:10.1103/PhysRevLett.113.078701) 25170736 10.1103/PhysRevLett.113.078701

[18] Ruths J, Ruths D 2014 Control profiles of complex networks. *Science* 343, 1373–1376. (doi:10.1126/science.1242063) 24653036 10.1126/science.1242063

[19] Wuchty S 2014 Controllability in protein interaction networks. *Proc. Natl Acad. Sci. USA* 111, 7156–7160. (doi:10.1073/pnas.1311231111) 24778220 10.1073/pnas.1311231111 PMC4024882

[20] Yuan ZZ, Zhao C, Wang WX, Di ZR, Lai YC 2014 Exact controllability of multiplex networks. *New J. Phys.* 16, 103036 (doi:10.1088/1367-2630/16/10/103036)

[21] Whalen AJ, Brennan SN, Sauer TD, Schiff SJ 2015 Observability and controllability of nonlinear networks: the role of symmetry. *Phys. Rev. X* 5, 011005 (doi:10.1103/PhysRevX.5.011005) 10.1103/PhysRevX.5.011005 PMC6234006 30443436

[22] Yan G, Tsekenis G, Barzel B, Slotine JJ, Liu YY, Barabási AL 2015 Spectrum of controlling and observing complex networks. *Nat. Phys.* 11, 779–786. (doi:10.1038/nphys3422)

[23] Chen YZ, Wang LZ, Wang WX, Lai YC 2015 The paradox of controlling complex networks: control inputs versus energy requirement. (http://arxiv.org/abs/1509.03196v1)

[24] Lin CT 1974 Structural controllability. *IEEE Trans. Automat. Contr.* 19, 201–208. (doi:10.1109/TAC.1974.1100557)

[25] Hopcroft JE, Karp RM 1973 An *n*^5/2^ algorithm for maximum matchings in bipartite graphs. *SIAM J. Comput.* 2, 225–231. (doi:10.1137/0202019)

[26] Zhou H, Ou-Yang ZC 2003 Maximum matching on random graphs. (http://arxiv.org/abs/cond-mat/0309348)

[27] Zdeborová L, Mézard M 2006 The number of matchings in random graphs. *J. Stat. Mech.* 2006, P05003 (doi:10.1088/1742-5468/2006/05/P05003)

[28] Hautus MLJ 1969 Controllability and observability conditions of linear autonomous systems. In *Nederlandse Academie van Wetenschappen. Series A: Mathematical Sciences*, vol. 72, pp. 443–448. Amsterdam, The Netherlands: Elsevier.

[29] Rugh WJ 1996 *Linear systems theory*. Englewood Cliffs, NJ: Prentice-Hall, Inc.

[30] Erdős P, Rényi A 1959 On random graphs, I. *Publ. Math.* 6, 290–297.

[31] Erdős P, Rényi A 1960 On the evolution of random graphs. *Publ. Math. Inst. Hung. Acad. Sci.* 5, 17–61.

[32] Sorrentino F, di Bernardo M, Garofalo F, Chen G 2007 Controllability of complex networks via pinning. *Phys. Rev. E* 75, 046103 (doi:10.1103/PhysRevE.75.046103) 10.1103/PhysRevE.75.046103 17500957

[33] Lai YC 2014 Controlling complex, nonlinear dynamical networks. *Nat. Sci. Rev.* 1, 339–341. (doi:10.1093/nsr/nwu023)

[34] Pasqualetti F, Zampieri S 2014 On the controllability of isotropic and anisotropic networks. In *53rd IEEE Conf. on Decision and Control*, pp. 607–612. Los Angeles, CA: IEEE.

